# The first report of hypohidrotic ectodermal dysplasia caused by a novel mutation and accompanied with pathological femoral neck fracture: A case report

**DOI:** 10.1097/MD.0000000000050191

**Published:** 2026-08-14

**Authors:** Guang-Hua Liang, Xiang-Ning Meng, Talante Juma, Yong-Ping Cao, Dao-Jian Zhang

**Affiliations:** a Department of Orthopedics, Peking University First Hospital, Beijing, China.

**Keywords:** femoral neck fracture, hypohidrotic ectodermal dysplasia, mutation, renal failure, *TP63*

## Abstract

**Rationale:**

Hypohidrotic ectodermal dysplasia (HED) is a rare inherited disorder characterized by hypohidrosis, hypotrichosis, and hypodontia. Most cases are caused by mutations in the EDA signaling pathway, whereas TP63-related HED is extremely rare. To our knowledge, this is the first reported case of HED caused by a novel TP63 mutation presenting with a pathological femoral neck fracture.

**Patient concerns:**

A 31-year-old woman presented with progressive left hip pain and inability to bear weight for 2 weeks without a history of trauma. She had a lifelong history of hypohidrosis, heat intolerance, sparse hair, hypodontia, dry skin, and nail abnormalities.

**Diagnoses:**

Physical examination and radiographs revealed a displaced femoral neck fracture. Laboratory investigations demonstrated severe anemia, end-stage renal disease, secondary hyperparathyroidism, vitamin D deficiency, and osteoporosis. Whole exome sequencing identified a previously unreported heterozygous *TP63* frameshift mutation (NM_001114982, c.1092_1093del, p.Asn364fs). Based on the clinical manifestations, laboratory findings, and genetic testing results, the patient was diagnosed with HED, pathological femoral neck fracture, end-stage renal disease, secondary hyperparathyroidism, osteoporosis, and severe anemia.

**Interventions:**

After correction of anemia and electrolyte imbalance by hemodialysis and blood transfusion, the patient underwent uncemented bipolar hemiarthroplasty.

**Outcomes:**

She began partial weight-bearing ambulation on postoperative day 3 and was discharged on postoperative day 6.

**Lessons:**

This case expands the mutational and phenotypic spectrum of *TP63*-associated HED by describing a previously unreported mutation presenting with a pathological femoral neck fracture and end-stage renal disease. It highlights the importance of early diagnosis, comprehensive genetic testing, multidisciplinary management, and regular follow-up for patients with HED.

## 1. Introduction

Ectodermal dysplasias (EDs) are genetic conditions affecting the development and/or homeostasis of 2 or more ectodermal derivatives; including hair, teeth, nails and certain glands like mammary, sweat and lacrimal glands.^[1]^ There are other ectodermal structures that can be involved, such as the anterior pituitary, thyroid glands, thymus and adrenal medulla.^[2]^ Among the 200 types of EDs that have been discovered, hypohidrotic or anhidrotic ectodermal dysplasia (HED/EDA) is the most frequent type of ED.^[3]^ HED can be differentiated from other types of ED by a triad of signs: a reduced or lack of ability to sweat, abnormal or lack of several teeth (hypodontia or anodontia), and sparse hair (hypotrichosis).

Although there are autosomal recessive and dominant forms, the X-chromosome-linked form is the most common type of HED; it is widely accepted that most of the X-chromosome-linked-ED phenotype is associated with mutations in the gene *EDA*, which encodes the transmembrane protein ectodysplasin-1 (EDA1) during the development of the fetus. *EDA* is a very important member of the TNFα-related signaling pathway; other genes in this pathway, such as *EDAR* and *EDARADD*, also regulate signal transduction from the ectoderm to mesenchyme, which is the most crucial step for the development of ectoderm-derived structures. Thus, any mutation in the genes related to this pathway could cause alterations in the initiation, formation, and differentiation of ectodermal appendages.

However, mutations in the *TP63* gene, which encodes the transcription factor p63, have been shown to play a significant role in ectodermal, orofacial, and limb development; *TP63* gene mutations rarely cause EDs compared to ectrodactyly-ectodermal dysplasia-cleft lip/palate syndrome (EEC syndrome), Acro-Dermato-Ungual-Lacrimal-Tooth (ADULT) syndrome and ankyloblepharon-ectodermal defects-cleft lip/palate syndrome (AEC syndrome).^[4]^ Due to the reduced penetrance and variable expression of the *TP63* gene, different combinations of these features can be observed in different cases.^[5]^

Femoral neck fractures (FNF) usually occur in elderly patients after they experiencemild trauma, while young patients are only observed to present with FNF after high-energy trauma or in those with poor bone quality, like renal osteopathy. The degrees of displacement and bone quality influence the prognosis of the internal fixation^[6]^; this is why surgeons prefer arthroplasty to internal fixation in patients with displaced FNF, regardless of their age. Here, we report a sporadic HED case with a novel mutation at a rare site of the *TP63* gene, which, to the best of our knowledge, has been previously unreported; this patient also had renal failure of unknown reason, which eventually leads to pathological FNF.

## 2. Case presentation

**Figure 1.**
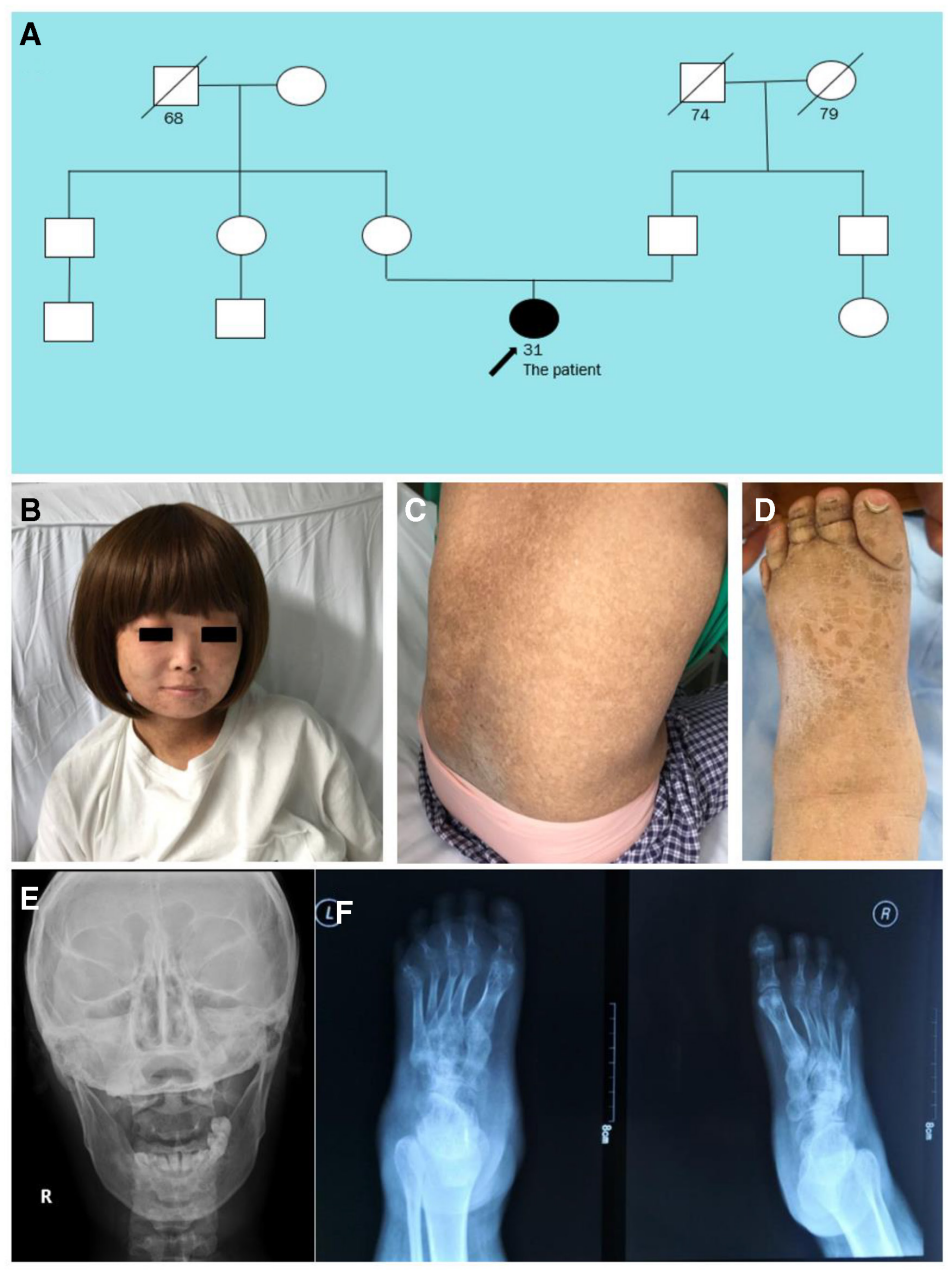
Comprehensive illustration of the patient’s clinical phenotypic features. (A) Pedigree chart. (B) Patient wearing a wig to conceal dry, thin, sparse hair, with a prominent forehead and saddle nose. (C) Fragile, dry skin with diffuse hyperpigmentation. (D) Gross appearance of the patient’s toes: dystrophic toenails; no acral deformities. (E) Rough, conical teeth with diastema and multiple missing teeth. (F) Anteroposterior foot radiograph.

The patient was a 31-year-old female, and she was the only child of unrelated, healthy Chinese parents. She presented with progressive left hip pain and inability to stand for 2 weeks with no obvious previous trauma. After reviewing the patient’s past medical record, we found that the patient was noted to have absence of sweating, intolerance to heat, and recurrent fever after birth. She also had dryness in nose and mouth but no disabilities of hearing and smell; she had been diagnosed with HED in a dermatology department over 20 years ago. None of her relatives, including direct relatives and collateral relatives up to 3 generations, has similar symptoms to hers (Fig. 1A showes the pedigree of her family), and she denied any other medical or medication history besides new emerging lower limb edema for 2 weeks.

A general physical examination revealed that the patient had dry, thin, and sparse hair on her scalp, a prominent forehead, a saddle nose, and normal lips with no cleft lip/palate (Fig. 1B). She only had several rough, conical, and wide-spaced teeth (Fig. 1E), and small coarse nails on her intact fingers and toes (Fig. 1D, F). She also had fragile, dry skin and hyperpigmentation all over her body with an absence of eyelashes and whole-body hair (Fig. 1C). She had no abnormalities of nipples or breast development. An orthopedic physical examination revealed a shortened and externally rotated left lower limb. The active flexion and the range of movement were dramatically limited, owing to her hip pain. The patient experienced particular pain upon axial loading and greater trochanter percussion of her hip.

**Figure 2.**
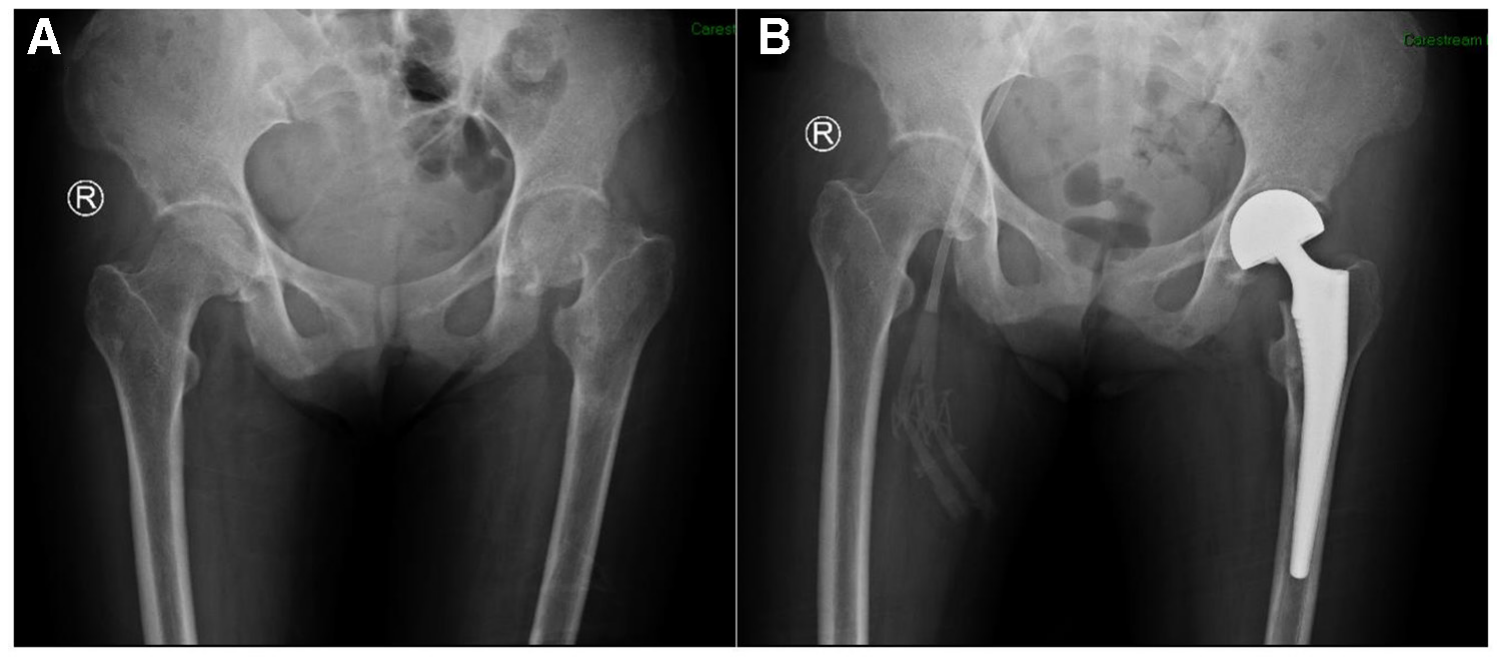
Preoperative (A) and postoperative (B) radiographs of the hip. The preoperative pelvic radiograph shows a displaced subcapital femoral neck fracture, and the postoperative image demonstrates a hemiarthroplasty.

After admission from the emergency department, we completed the standard preoperative tests and examinations. The laboratory evaluations showed a severe, anemic hemoglobin level of 49 g/L (reference range [RR]: 115–150 g/L) and end-stage renal failure with a highly elevated serum creatinine level of 600.60 μmol/L (RR: 44–133 μmol/L). The calcium and phosphate levels were 1.54 mmol/L (RR: 2.11–2.52 mmol/L) and 1.74 mmol/L (RR: 0.85–1.51 mmol/L), respectively, and the parathyroid hormone (PTH) level was 176.60 pg/mL (RR: 15–65 pg/mL), which was suggestive of secondary hyperparathyroidism. The serum vitamin D (25-OH-VitD) level was 21.19 nmol/L (RR: 75–250 nmol/L), which was severely insufficient. The serum magnesium level was 1.20 mmol/L (RR: 0.75–1.02 mmol/L), and the potassium level was 5.41 mmol/L (RR: 3.5–5.3 mmol/L). Pelvic x-rays revealed a displaced subcapital FNF (Fig. 2A). However, the other tests and examinations, including the tumor marker levels and abdominal ultrasonography, were unremarkable.

Of note, this patient did not know about her renal failure prior to the fracture, as she reported that her renal function was normal 5 years ago. She also denied any drug use, urinary symptoms, and edema of the lower limbs except for the recent 2 weeks. To handle this case, we formed a small multidisciplinary team that included orthopedic surgeons, a nephrologist, and a dermatologist. The nephrologist also could not confirm the cause of the development of the renal failure but urgently recommended starting hemodialysis, which was quickly arranged after the catheterization of the femoral vein. The dermatologist reconfirmed the diagnosis of HED and suggested the completion of whole exome sequencing (WES), which surprisingly showed a novel mutation in the *TP63* gene (NM_001114982, c.1092_1093del, p.Asn364fs). Common gene variants usually seen in HED like, *EDA*, *EDAR*, *EDARADD*, *WNT10A*, *CHD3*, *KRT85* and *IKBKG*, were also favored in the filtering process of WES. According to the report of WES, *TP63* was located on the third chromosome q28 with an autosomal dominant manner; the mutation sequence was NM_001114982 and it caused a deletion of nucleotides in c.1092_1093, which had rarely been reported before. Combining her medical history and laboratory parameters, she was diagnosed with a left femoral neck fracture (Garden III), end-stage renal failure, secondary hyperparathyroidism, HED, osteoporosis and severe anemia.

After 2 rounds of hemodialysis and blood transfusion, we were able to quickly correct the severe anemia and water-electrolyte disorder. Considering the subcapital displaced fracture with a high risk of causing avascular necrosis and a poor outcome for internal fixation because of the severe osteoporosis secondary to renal failure, an elective surgery of uncemented bipolar hemi-hip arthroplasty was performed. Postoperative x-rays were taken (Fig. 2B), and the patient was able to start walking with partial weight-bearing on the third day post-operation. She was discharged on the sixth day post-operation with anticoagulants, anti-osteoporotic supplements, and advice for 3 times weekly dialysis. The dermatologist’s recommendations for the HED were the avoidance of overheating, external use of cetirizine, and the application of creams to protect the dry and delicate skin from possible damage.

## 3. Discussion

HED is a very rare group of disorders that mainly cause defects in structures derived from the ectoderm; it occurs in the general population with an incidence of 1/17,000 live births.^[7,8]^ At present, there is no acknowledged classification or genotype–phenotype correlation for patients with HED. There are very few HED cases that are caused by mutations in the *TP63* gene, as most of the HED mutations are found in the *EDA, EDAR*, and *EDARADD* genes. However, we only found a single-digit number of cases from the available literature in which a *TP63* gene mutation resulted in HED without ectrodactyly, cleft lip, or ankyloblepharon; whereas, *TP63* gene mutations often cause EEC, ADULT, and AEC syndromes.^[9]^

The WES results and most of the relevant literature showed that inherited *TP63* gene mutations are usually in an autosomal dominant manner, and only approximately 30% of *TP63* mutated individuals have an affected parent, while approximately 70% of the patients have a de novo pathogenic variant.^[10,11]^ In our case, all of the patient’s relatives including her parents and collateral relatives up to 3 generations were all normal, so we assumed it was a de novo mutation. According to the guidelines of D.G. MacArthur and the American College of Medical Genetics and Genomics (ACMG), the *TP63* (NM_001114982, c.1092_1093del, p.Asn364fs) variant belongs to VUS 1 (Variants of Uncertain Significance type 1), which is considered a likely pathogenic variant.^[12,13]^ It has been reported that *TP63* knockout heterozygous mice showed defects in the skin, oral epithelium, mammary glands, limbs, and craniofacial regions, while *TP63* knockout mice showed severe developmental anomalies, including truncated forelimbs and the absence of hind limbs with a high death rate.^[14]^ Besides, missense and insertion mutations in *TP63* are common types of mutations in the disease mechanism, but a deletion mutation in *TP63* was observed in this case study,^[15,16]^ which could explain the differential expression of *TP63* caused by the novel mutation in our case. Usually, nails are normal in HED patients with *EDA/EDAR/EDARADD* mutations, while hyponychia is well known in *WNT10A* mutated patients.^[17]^ In *TP63* mutation-related disorders, nails are clearly and very frequently abnormal, which is consistent with this case.^[18]^

In spite of *TP63* gene mutations being reported in correlation with urinary system deformities that could lead to end-stage renal disease, we still could not explain the reason for this patient’s renal failure, so did the renal physician, as her abdominal ultrasonography showed no anomalies and she had no symptoms related to the urinary system until 2 weeks ago.^[19]^ We assumed that there might be some hidden medical history regarding drug use or urinary symptoms. Otherwise, the renal failure is associated with gene mutations, as part of this syndrome, by mechanisms that we have not yet discovered. So, in the future, we are planning to study the effects of *TP63* on bone density and renal function in *TP63* knockout mice. Nevertheless, there is no doubt about the importance of early diagnosis and an awareness of the variable phenotypes observed in HED cases. In addition, we recommend a yearly general checkup for HED patients in order to facilitate the early detection of any possible abnormalities.

In general, the preservation of the natural hip anatomy and mechanics is a priority in young patients with FNF, due to their high functional demands and the possibility of subsequent revision surgery.^[20]^ But in our case, we performed arthroplasty instead of internal fixation due to the high occurrence rate of avascular necrosis and bone nonunion in subcapital FNF. We chose uncemented hemi-hip arthroplasty instead of total hip arthroplasty because of the uncertainty of the patient’s life-span, due to HED and the complications associated with long-term hemodialysis; the patient’s very low amount of physical activity, as she rarely goes out; low success rate of cemented prosthetic fixation in severe osteoporosis patients with high bone turnover rate; and the poor economic condition of the patient’s family.

## 4. Conclusion

By presenting a sporadic HED case that had a novel mutation at a rare site of *TP63*, and thoroughly reviewing related literature, we provided a new mutation of HED and raised the question of whether *TP63* has an effect on the bone and urinary system. Besides, as many phenotypes and mutations of HED have been discovered over time, we should be aware of the fact that other gene mutations may cause the same phenotypes by affecting the same or different pathways, and furthermore, the same mutation could be expressed in variable phenotypes. Along with the accumulation of HED cases, additional steps should be taken to classify HED in detail and to study the possible pathways of HED.

## Acknowledgments

We thank the patient and her family members for their participation in the study. We are grateful to the clinical lab for the WES.

## Author contributions

**Conceptualization:** Talante Juma, Yong-Ping Cao, Dao-Jian Zhang.

**Data curation:** Xiang-Ning Meng.

**Methodology:** Yong-Ping Cao.

**Project administration:** Talante Juma, Yong-Ping Cao.

**Validation:** Xiang-Ning Meng.

**Writing – original draft:** Guang-Hua Liang.

**Writing – review & editing:** Guang-Hua Liang.
